# Histone Methylations Define Neural Stem/Progenitor Cell Subtypes in the Mouse Subventricular Zone

**DOI:** 10.1007/s12035-019-01777-5

**Published:** 2019-10-25

**Authors:** Zhichao Zhang, Adeel Manaf, Yanjiao Li, Sonia Peña Perez, Rajikala Suganthan, John Arne Dahl, Magnar Bjørås, Arne Klungland

**Affiliations:** 1 Institute of Neurobiology, Xi’an Jiaotong University Health Science Center, Xi’an, China; 2 Department of Microbiology, Oslo University Hospital, Rikshospitalet, Sognsvannsveien 9, 0372 Oslo, Norway; 3 Department of Molecular Medicine, Institute of Basic Medical Sciences, University of Oslo, Oslo, Norway; 4 Department of Clinical and Molecular Medicine, Norwegian University of Science and Technology, Høgskoleringen 1, 7491 Trondheim, Norway

**Keywords:** Histone methylation, NSPC subtypes, Mouse subventricular zone, Neurodevelopment

## Abstract

Neural stem/progenitor cells (NSPCs) persist in the mammalian brain throughout life and can be activated in response to the physiological and pathophysiological stimuli. Epigenetic reprogramming of NPSC represents a novel strategy for enhancing the intrinsic potential of the brain to regenerate after brain injury. Therefore, defining the epigenetic features of NSPCs is important for developing epigenetic therapies for targeted reprogramming of NSPCs to rescue neurologic function after injury. In this study, we aimed at defining different subtypes of NSPCs by individual histone methylations. We found the three histone marks, histone H3 lysine 4 trimethylation (H3K4me3), histone H3 lysine 27 trimethylation (H3K27me3), and histone H3 lysine 36 trimethylation (H3K36me3), to nicely and dynamically portray individual cell types during neurodevelopment. First, we found all three marks co-stained with NSPC marker SOX2 in mouse subventricular zone. Then, CD133, Id1, Mash1, and DCX immunostaining were used to define NSPC subtypes. Type E/B, B/C, and C/A cells showed high levels of H3K27me3, H3K36me3, and H3K4me3, respectively. Our results reveal defined histone methylations of NSPC subtypes supporting that epigenetic regulation is critical for neurogenesis and for maintaining NSPCs.

**Electronic supplementary material:**

The online version of this article (10.1007/s12035-019-01777-5) contains supplementary material, which is available to authorized users.

## Introduction

**Fig. 1:**
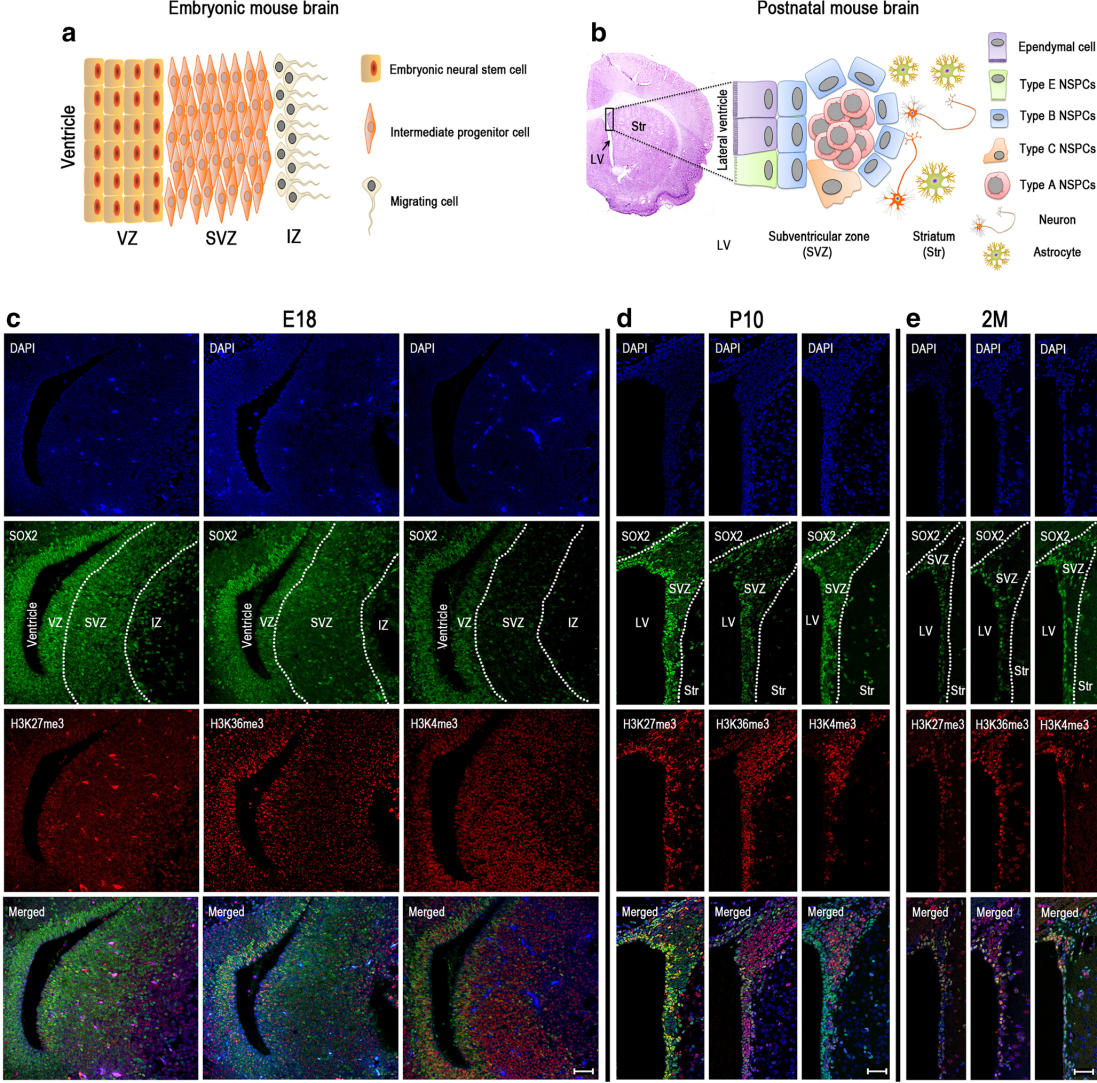
H3K27me3, H3K36me3, and H3K4me3 co-located with SOX2 during neurodevelopment in SVZ. Schematics of the cell layers and cell types in the embryonic (**a**) and adult (**b**) brain. Immunofluorescent staining showed that high level of H3K27me3, H3K36me3, and H3K4me3 co-stained with SOX2 at E18 (**c**), P10 (**d**), and 2M (**e**). Nuclei were counterstained with DAPI. E18, embryo at day 18; P10, postnatal at day 10; 2M, adults 2 months. Scale bar = 50 μm

In the postnatal mammalian brain, most of the neural stem/progenitor cells (NSPCs) are spatially restricted to two specific brain regions: the subgranular zone (SGZ) in the dentate gyrus of the hippocampus and the subventricular zone (SVZ) of the lateral ventricles [1]. As the major site for NSPCs in the postnatal central nervous system (CNS), four major cell types of NSPCs have been identified in the SVZ niche: ependyma-like stem NSPCs (type E cells), quiescent or dormant NSPCs (qNSCs; type B cells), transient amplifying progenitors (TAPs; type C cells), and migrating neuronal precursors (neuroblasts; type A cells) [2, 3] (Fig. 1b). NSPCs in SVZ can be activated in response to physiological and pathophysiological stimuli, in which they initiate CNS repair and functional recovery [4]. Therefore, understanding the dynamic regulation of NSPC subtypes may provide new insight for developing novel treatment modalities for CNS diseases.

Histone modifications are post-translational modifications to histone proteins which include methylation, phosphorylation, acetylation, ubiquitylation, and sumoylation. These modifications have biological roles and can be inherited and are referred to as epigenetic marks. Specific histone methylation marks at promoter regions affect transcription activities [5]. Generally, histone H3 lysine 4 trimethylation (H3K4me3) and histone H3 lysine 36 trimethylation (H3K36me3) are associated with active promoters and gene bodies of actively transcribed genes. This results in increased transcription activity, whereas histone H3 lysine 27 trimethylation (H3K27me3) is linked to transcriptional repression [6]. H3K4me3, H3K36me3, or H3K27me3 has pivotal and distinct roles in different stages of neurodevelopment and aberrant regulation of histone methylation contributes to the pathogenesis of various CNS disorders [7]. Many embryonic stem cell (ESC) promoters combine activating H3K4me3 marks and repressive H3K27me3 marks, and these bivalent domains are important dynamically regulated targets in the expression of developmental genes [8]. H3K36me3 is markedly enriched at pericentromeric heterochromatin in ESCs and fibroblasts [9]. Even though both H3K4me3 and H3K36me3 are transcriptional activators, H3K36me3 predominates in the transcribed bodies of genes, whereas nucleosomes near the transcription start site of active genes contain H3K4me3 [10]. However, we have limited understanding regarding the function of the dynamic changes in these histone methylation marks during neurodevelopment.

In this study, we observed distinct features of histone methylation in the different subtypes of NSPCs during neurodevelopment. Type E/B cells are marked by high levels of H3K27me3, type B/C cells showed high levels of H3K36me3, and H3K4me3 is specific for type C/A cells. These results may reveal new insight into the onset of neurodevelopment and provide an innovative epigenetic signature for discovery and characterization of key regulatory genes/regions for neurogenesis.

## Material and Methods

### Animals

C57BL/6N mouse strain was used for this research and all mouse experiments were approved by the Animal Research Committee and the Norwegian Food Safety Authority (NFDA), and conducted in accordance with the rules and regulations of the Federation of European Laboratory Animal Science Associations (FELASA). The staff at Komparativ Medisin (KPM) Oslo University Hospital is responsible for housing and daily maintenance. Housing and environmental enrichment is according to standards. All efforts were made to minimize animal suffering and to keep the numbers of animals used to a minimum.

### Method Details

P10 and adult mice were anesthetized and transcardially perfused with normal saline followed by 4% paraformaldehyde (PFA, sc-281692, Santa Cruz Biotechnology, Dallas, TX, USA). Ten milliliter normal saline and 25 ml 4% PFA were used for P10 mice, while adult mice were infused with 25 ml normal saline and 50 ml 4% PFA. For E18 mice, pregnant E18 mice were sacrificed and the fetal brains were dissected in cold PBS, and then soaked into 4% PFA for fixation. All brains were dissected and post-fixed in 4% PFA overnight at 4 °C, followed by paraffin embedding. Four-micrometer brain tissue serial slices were coronally sectioned by microtome (HM355s, Thermo Scientific, Waltham, MA, USA) and mounted onto glass slides. These sections were used for immunostaining. The slides were deparaffinized and cleared in Clear-Rite™ 3 (6901TS, Thermo Scientific) followed by rehydration in an EtOH gradient. Then the slides were heated to 95 °C in the antigen retrieval buffer (3 g sodium citrate (25114, Sigma-Aldrich), 0.4 g citric acid (251275, Sigma-Aldrich), 1000 mL H_2_O, pH 6.0) for 30 min, followed by washing with 0.01 M PBS (all washes were performed three times, 5 min each). The slides were permeabilized with 0.3% Triton X-100 (T8787, Sigma-Aldrich, St. Louis, MO, USA) for 20 min, rinsed, and then blocked for 2 h with blocking buffer (5% normal goat serum (G9023, Sigma-Aldrich) and 5% bovine serum albumin (A7096, Sigma-Aldrich)). The samples were incubated with the primary antibodies (Table 1) overnight at 4 °C, washed with 0.01 M PBS, and then incubated with the suitable secondary antibodies. All antibodies are shown in Key resources table. The control samples were incubated in blocking buffer instead without primary antibodies. Nuclei were visualized with mounting medium including DAPI (H-1200, Vector, Burlingame, CA, USA). Images were taken with a Leica SP8 confocal microscope equipped with a × 40 oil immersion lens. The number of immunolabeled cells lining the lateral wall of the lateral ventricle was counted for three sections in each mouse, and at least three animals were used for each experiment.

**Table 1:** Antibodies used during the study

Antigen	Source and host species	Concentration	Catalog no.
Anti-SOX2	Abcam, mouse monoclonal antibody	1:200	ab79351
Anti-SOX2	Abcam, rabbit polyclonal antibody	1:200	ab97959
Anti-Ki67	Abcam, rabbit polyclonal antibody	1:200	ab15580
Anti-Ki67	Invitrogen, rat monoclonal antibody	1:1000	14-5698-80
Anti-PCNA antibody	Abcam, rabbit monoclonal antibody	1:1000	ab29
Anti-PCNA antibody	Abcam, mouse monoclonal antibody	1:200	ab92552
Anti-CD133 antibody	Millipore, rat monoclonal antibody	1:100	MAB4310
Anti-ID1 antibody	R&D Systems, goat polyclonal antibody	1:200	AF4377
Anti-MASH1 antibody	Abcam, rabbit monoclonal antibody	1:1000	ab213151
Anti-MASH1 antibody	BD Pharmingen, mouse monoclonal antibody	1:100	556604
Anti-DCX antibody	Abcam, rabbit polyclonal antibody	1:200	ab18723
Anti-DCX antibody	BD Transduction Laboratories, mouse monoclonal antibody	1:200	611706
Anti-trimethyl-Histone H3 (Lys4) (H3K4me3) antibody	Millipore, rabbit monoclonal antibody	1:500	04-745
Anti-Histone H3 trimethyl Lys36 (h3k36me3) antibody	Active Motif, mouse monoclonal antibody	1:500	61021
Anti-Histone h3k27me3 (H3K27 Trimethyl) antibody	Epigentek, rabbit Polyclonal antibody	1:150	A-4039
Anti-Mouse igg (H+L) secondary antibody, Alexa Fluor 488	Invitrogen, donkey polyclonal antibody	1:500	R37114
Anti-Mouse IgG (H+L) secondary antibody, Alexa Fluor 594	Invitrogen, donkey polyclonal antibody	1:500	R37115
Anti-Rabbit IgG (H+L) secondary antibody, Alexa Fluor 488	Invitrogen, donkey polyclonal antibody	1:500	R37118
Anti-Rabbit IgG (H+L) secondary antibody, Alexa Fluor 594	Invitrogen, donkey polyclonal antibody	1:500	R37119
Anti-Rat IgG (H+L) Highly Cross-Adsorbed secondary antibody, Alexa Fluor 594	Invitrogen, donkey polyclonal antibody	1:500	A-21209
Anti-Goat IgG (H+L) Cross-Adsorbed secondary antibody, Alexa Fluor 594	Invitrogen, donkey polyclonal antibody	1:500	A-11058

### Quantification and Statistical Analysis

The level of histone methylation and double-positive cell was measured and defined by using Image-Pro Plus 5.1. Differences between groups were analyzed using one-way ANOVA, followed by Tukey’s post hoc test. All statistical analyses were performed using GraphPad Prism 5. The data are shown as mean ± standard deviation, and *P* < 0.05 was considered as statistically significant difference.

## Results

### High Levels of H3K27me3, H3K36me3, and H3K4me3 in Neural Stem/Precursor Cells during Neurodevelopment in SVZ

To characterize the dynamics of histone methylations during neurodevelopment, we collected mouse brains at different time points of early life: embryo at day 18 (E18), postnatal at day 10 (P10), and adults at 2 months (2M). Then, we examined the levels of three different histone methylation marks (H3K27me3, H3K36me3, and H3K4me3) by immunofluorescence staining. All three histone marks showed strongest staining in neurogenic niches (e.g., SVZ and SGZ) during neurodevelopment, although the intensity varied among the three time points. In SVZ, the three tested histone marks showed co-localization with the established NSPC marker SOX2 at all three time points of development studied (Fig. 1c–e). In SGZ, SOX2 co-localized with H3K4me3 and H3K36me3 at all three time points, while H3K27me3 SOX2 double-positive cells were just sporadic at any time point (Supplement Fig. 1 A–D). Notably, H3K4me3, H3K36me3, and H3K27me3 stained distinct parts of SVZ, particularly at P10. H3K27me3 showed strongest staining of the ependymal cell layer, and H3K36me3 level was high in the surrounding striatal parenchyma as well as the ependymal cell layer at the lateral ventricle. In contrast, H3K4me3 staining is strongest between the ependymal cell layer and the striatal parenchyma (Fig. 1d). Previously, it has been demonstrated that in the postnatal mouse brain, type B cells locate between type A cells and the underlying striatal parenchyma as well as between type A cells and the ependymal cells, and that type C cells locate around type A cells (Fig. 1b) [11, 12]. These results suggest that histone methylation may define different subtypes of NSPCs.

### High Level of H3K27me3 in CD133-Positive Cells at Early Postnatal Neurodevelopment

**Fig. 2:**
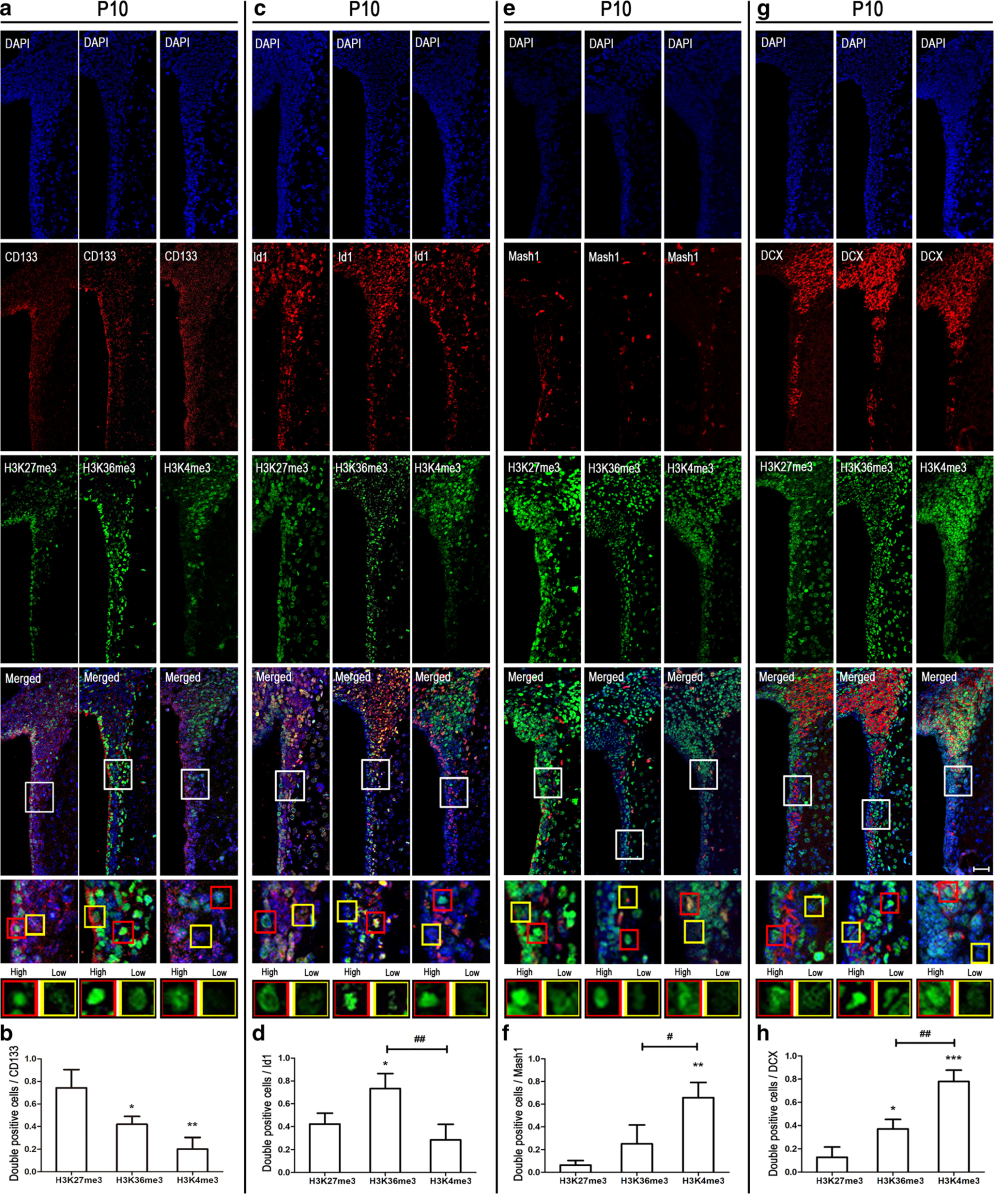
Different subtypes of NSPC marker co-stained with different histone methylations at postnatal 10 days (P10). Immunocytochemical double labeling revealed different patterns of histone methylations co-stained with CD133 (**a**), Id1 (**c**), Mash1 (**e**), and DCX (**g**) respectively. Nuclei were counterstained with DAPI. Scale bar = 50 μm. The square frames are enlarged to identify a single typical high (red) and low (yellow) level cell relating to the different histone methylation mark. **b**, **d**, **f**, **h** The number of immunolabeled cells was counted for three sections in each mouse and each value represents the mean ± SD of three mice (*n* = 3). *P < 0.05, **P < 0.01, ***P < 0.001 versus H3K27me3 group; ^#^*P* < 0.05, ^##^*P* < 0.01 versus H3K36me3 group. P10, postnatal at day 10

**Fig. 3:**
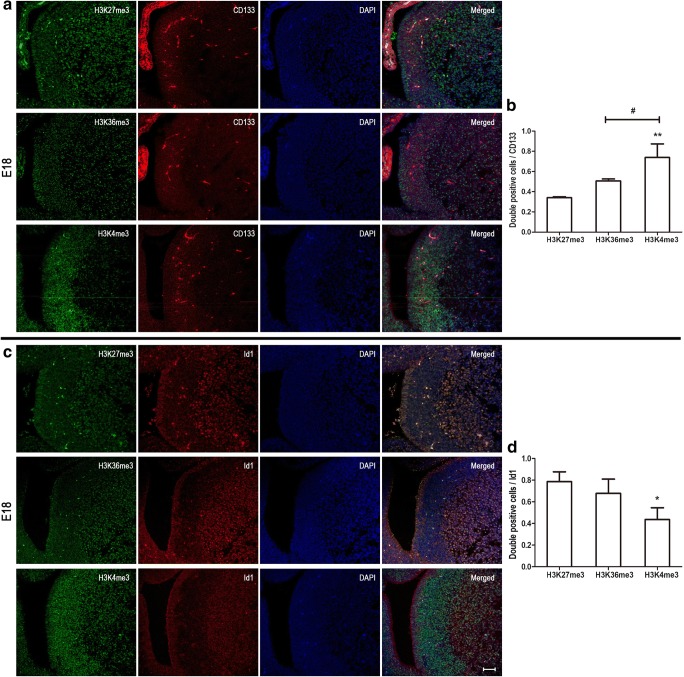
CD133 and Id1 co-stained with different histone methylations at E18. Immunocytochemical double labeling revealed different patterns of histone methylations co-stained with CD133 (**a**) and Id1 (**c**), respectively. Nuclei were counterstained with DAPI. Scale bar = 50 μm. **b**, **d** The number of immunolabeled cells was counted for three sections in each mouse and each value represents the mean ± SD of three mice (*n* = 3). *P < 0.05, **P < 0.01 versus H3K27me3 group; #*P* < 0.05 versus H3K36me3 group. E18, embryo at day 18

In postnatal mouse brain, CD133 (also known as prominin-1) is a marker for type E/B cells; Id1 marks type B/C cells (type C cells are Id1 positive, although at significantly lower levels relative to type B cells) [13]; type C cells express the highest levels of Mash1 (also known as Ascl1); and DCX marks type A cells [14]. Immunocytochemical double labeling unveils 74% CD133-positive cells showing high level of H3K27me3. On the contrary, there were few CD133-positive cells that co-stained with H3K36me3 (42%) and H3K4me3 (20%) (Fig. 2a, b). In the adult SVZ, the number of CD133-positive cell decreased markedly and there was no significant difference in immunocytochemical double labeling for the three histone methylation marks studied at this stage (Fig. 5a, b). The anatomical structure of embryo and postnatal mouse brain is noticeably different (Fig. 1a, b). In embryo mouse brain, H3K27me3 and H3K36me3 showed high levels in the ventricular zone (VZ), while high levels of H3K4me3 cells were located to SVZ (Fig. 1c). As expected, most cells in VZ and SVZ were CD133 positive. Furthermore, 74% of the CD133-positive cells co-stained with H3K4me3, while H3K27me3 and H3K36me3 showed 34% and 51% co-staining with CD133, respectively (Fig. 3a, b). Thus, it appears that high level of H3K27me3 is displayed in ependymal and quiescent neural stem cells in the SVZ (type B/E) at early postnatal neurodevelopment.

### High Level of H3K36me3 in Id1-Positive Cells at Early Postnatal Neurodevelopment

Similarly, we used immunocytochemical double labeling to identify colocalization of Id1 and the three histone methylation marks. Seventy-three percent Id1-positive cells co-stained with H3K36me3, significantly higher than H3K27me3 (42%) and H3K4me3 (29%) (Fig. 2c, d). Analogous to the CD133 staining, the number of Id1-positive cells was reduced dramatically at adulthood and double labeling revealed minor differences for the three histone methylation marks (Fig. 5c, d). In embryo mouse brain, most of the Id1-positive cells were located in VZ and the majority of Id1-positive cells co-stained with H3K27me3 (79%) and H3K36me3 (68%) (Fig. 3c, d). However, just 44% H3K4me3-positive cells co-stained with Id1. These phenomena may indicate that H3K36me3 is a good marker for quiescent and active neural stem cells (type B/C) at early postnatal neurodevelopment.

### High Level of H3K4me3 in Mash1 and DCX-Positive Cells at Postnatal Neurodevelopment

**Fig. 4:**
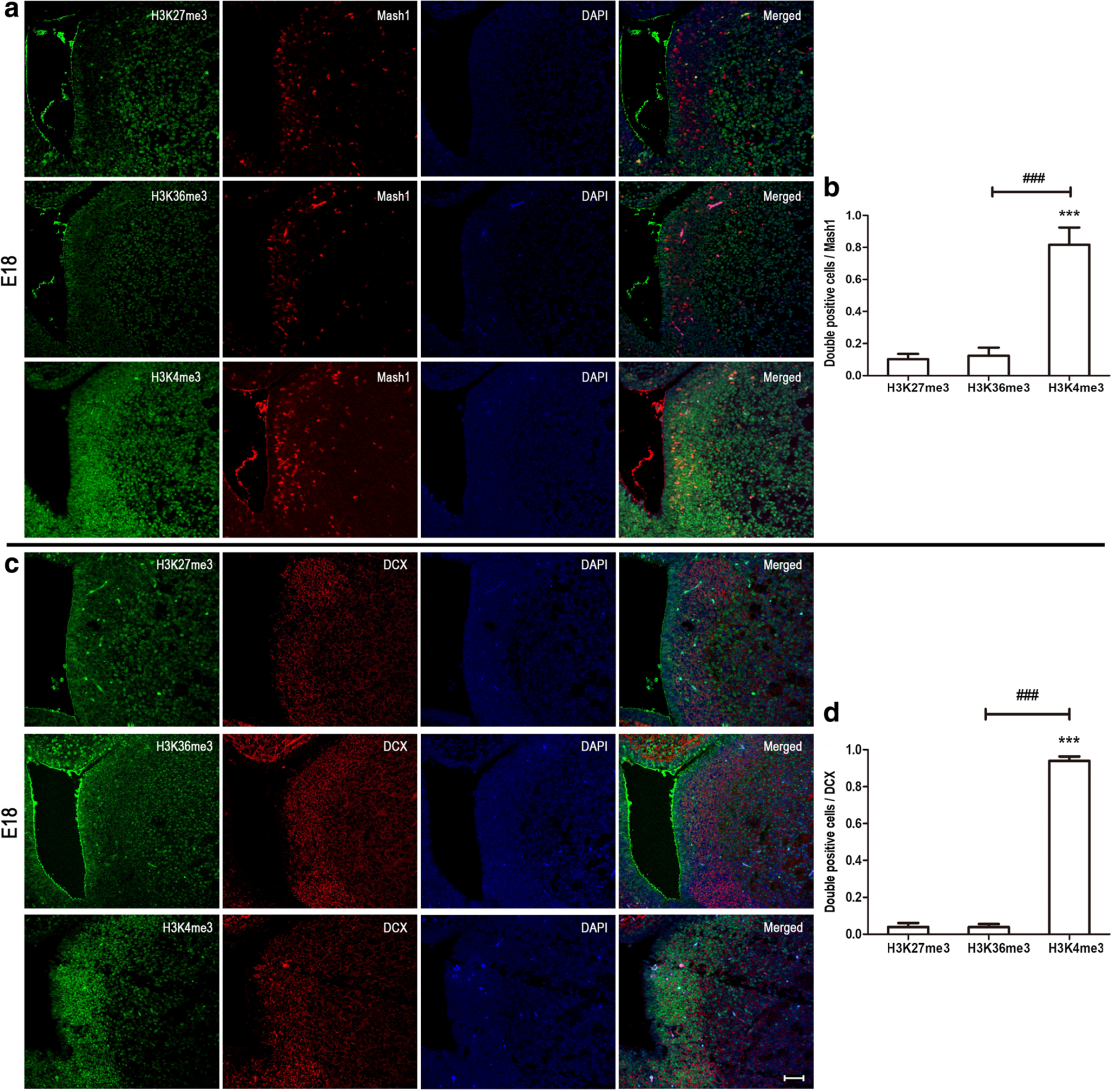
Mash1 and DCX co-stained with different histone methylations at E18. Immunocytochemical double labeling showed different patterns of histone methylations in Mash1- (**a**) and DCX (**c**)-positive cells. Nuclei were counterstained with DAPI. Scale bar = 50 μm. **b**, **d** The number of immunolabeled cells was counted for three sections in each mouse and each value represents the mean ± SD of three mice (*n* = 3). ***P < 0.001 versus H3K27me3 group; ###*P* < 0.001 versus H3K36me3 group. E18, embryo at day 18

**Fig. 5:**
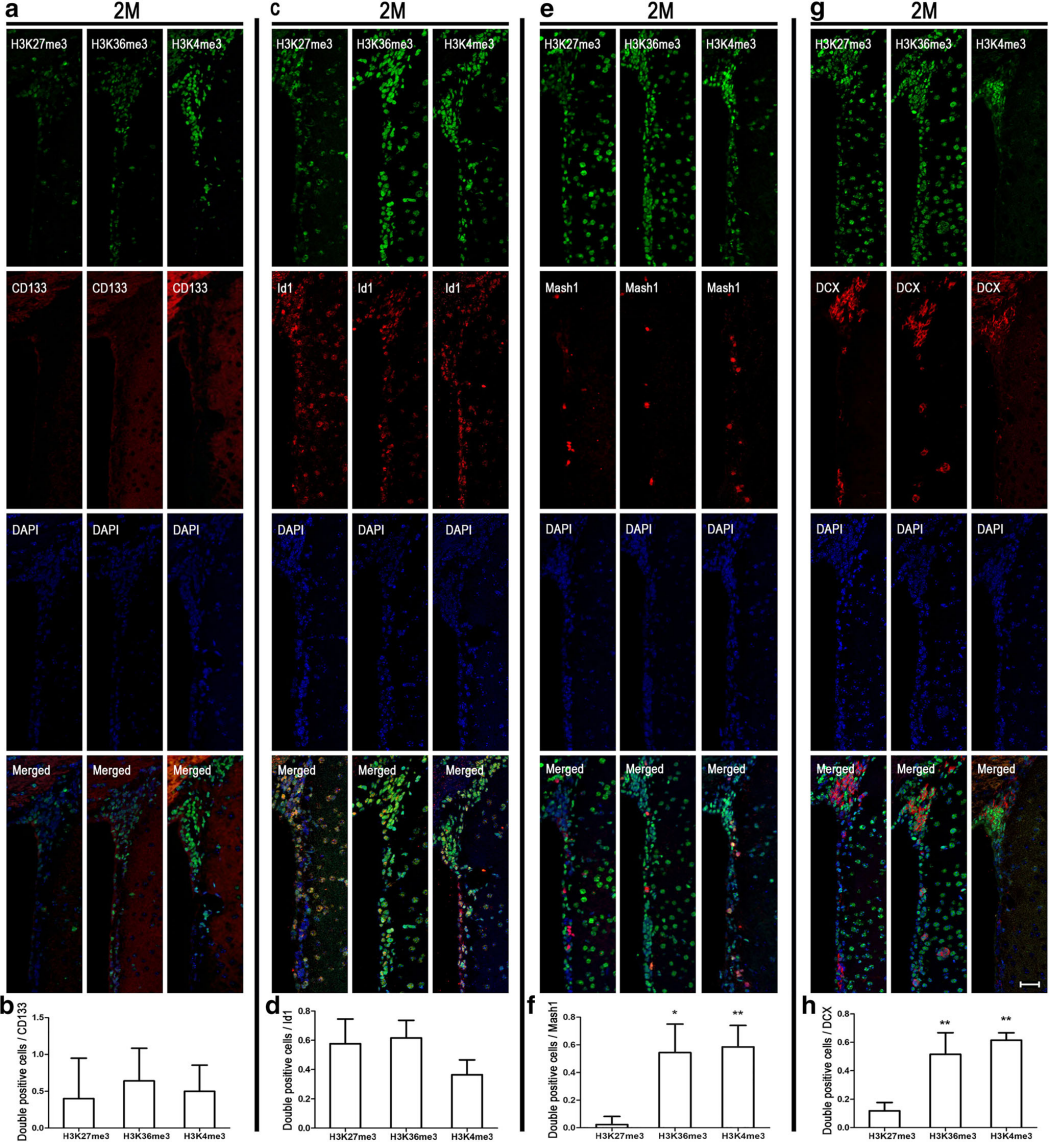
Different subtypes of NSPC marker co-stained with different histone methylations at 2 months. Immunocytochemical double labeling showed different patterns of histone methylations co-stained with CD133 (**a**), Id1 (**c**), Mash1 (**e**), and DCX (**g**), respectively. Nuclei were counterstained with DAPI. Scale bar = 50 μm. **b**, **d**, **f**, and **g** The number of immunolabeled cells was counted for three sections in each mouse and each value represents the mean ± SD of three mice (*n* = 3). *P < 0.05, **P < 0.01 versus H3K27me3 group. 2M, 2 months

Mash1 (also known as Ascl1) is characterized as a proneural transcription factor and typically used as a type C cell marker. DCX is expressed in the last stage before NSPCs are migrating through the rostral migratory stream (RMS) [14]. Therefore, Mash1 and DCX were used for labeling type C and A cell, respectively. Immunocytochemical double labeling identified 66% of Mash1-positive cells co-staining with H3K4me3 at P10, while very low co-staining was observed for H3K27me3 (6%) and H3K36me3 (25%) (Fig. 2 E, F). Embryonic brain staining results showed that Mash1-positive cell appeared in SVZ; and it was similar to P10 with 82% H3K4me3, and very low H3K27me3 and H3K36me3 co-staining, 10% and 13% respectively (Fig. 4a, b). At adulthood, 58% Mash1-positive cells co-stained with H3K4me3, 2% with H3K27me3, and 54% with H3K36me3 (Fig. 5e, f). Then, double immunostaining was also used for detecting DCX and different histone methylations. During neurodevelopment, the number of H3K4me3 DCX double-positive cells was significantly higher compared with H3K27me3 or H3K36me3 double-positive cells (Fig. 2g, h; Fig. 4c, d; and Fig. 5g, h). Thus, both type C and type A are represented by H3K4me3.

### H3K4me3- and H3K36me-Positive Cells Co-Stain with Proliferation Markers at Early Neurodevelopment

**Fig. 6:**
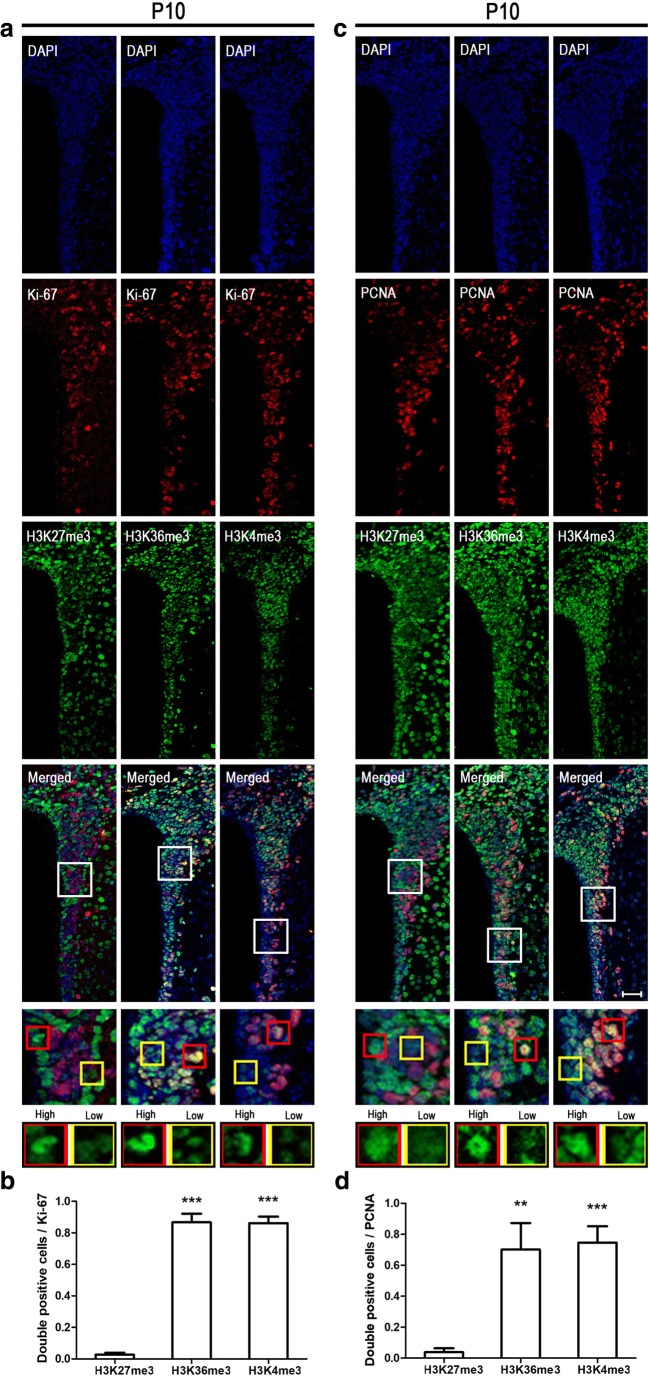
High levels of H3K4me3 and H3K36me3 in cells expressing Ki-67 and PCNA at postnatal 10 days (P10). Immunocytochemical double labeling showed different patterns of histone methylations in Ki-67- **(a**) and PCNA (**c**)-positive cells. Nuclei were counterstained with DAPI. Scale bar = 50 μm. The square frames are enlarged to show the typical detail high (red) and low (yellow) levels of different histone methylation features. **b**, **d** The number of immunolabeled cells was counted for three sections in each mouse and each value represents the mean ± SD of three mice (*n* = 3). **P < 0.01, ***P < 0.001 versus H3K27me3 group. P10, postnatal at day 10

To further evaluate histone methylation in the proliferation state of early developmental cells in SVZ, co-staining with the proliferation markers Ki-67 and PCNA was analyzed. We identified a noticeable difference with most of Ki-67-positive cells co-staining with H3K36me3 (87%) or H3K4me3 (86%), while only 3% of H3K27me3-positive cells co-stained with Ki-67 at P10 (Fig. 6a, b). Similarly, just 4% of PCNA-positive cells co-stained with H3K27me3 compared with 70% for H3K36me3 and 75% for H3K4me3 (Fig. 6c, d). These results strongly indicate that high levels of H3K36me3 and H3K4em3 correlate very well with proliferating cells in SVZ at early postnatal neurodevelopment.

## Discussion

**Fig. 7:**
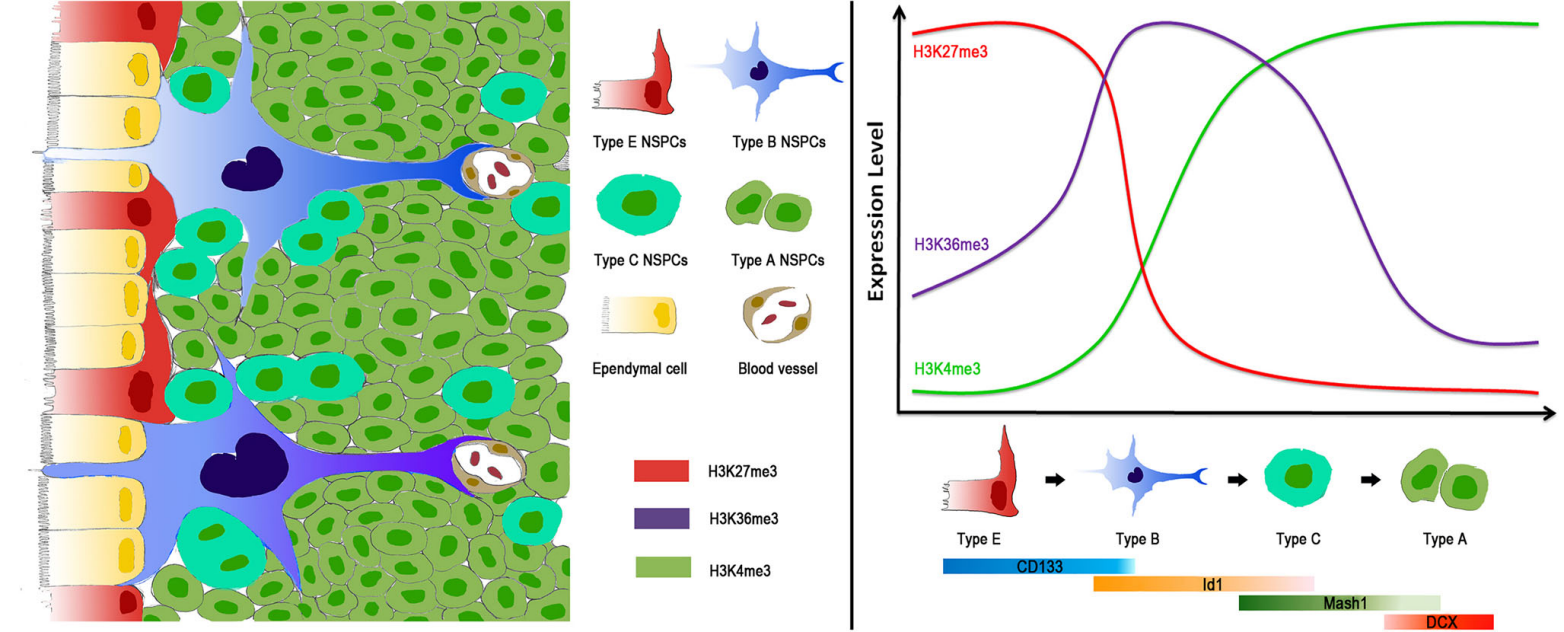
Schematic model of the developmental process of NSPCs projected from this study. Histone methylations are dynamically changed during NSPC differentiation in mouse SVZ area. Different subtypes of NSPCs represented different patterns of histone methylations. Specifically, type E/B cells are marked by high levels of H3K27me3, type B/C cells showed high levels of H3K36me3, and H3K4me3 is specific for type C/A cells

Traditional therapies for CNS diseases are limited. For example, treatment for clinical stroke by the administration of tissue plasminogen activator and the recent introduction of mechanical thrombectomy can only be used in a limited proportion of patients due to time constraints [15]. Accordingly, continuing efforts are in need to develop novel, safe, and more optimal and effective therapeutic strategies for CNS diseases. The dynamic regulation of histone methylations and chromatin remodeling plays essential roles in development, cellular differentiation, and cell fate maintenance [16]. More importantly, emerging evidence supports the involvement of histone methylation in the pathogenesis of CNS damage and several neurodegenerative diseases [17, 18]. In this study, we reveal how different histone methylation marks are dynamically regulated during NSPC differentiation in the mouse SVZ area, represented as marked differences in histone methylations between quiescent and active NSPCs (Fig. 7). As NSPCs can be activated by CNS damage and participate in CNS repair and functional recovery, our study may bring a novel perspective to a therapeutic strategy of CNS diseases and provide a potential histone methylation features for screening and identifying key therapeutic genes for CNS diseases.

SOX2 maintain stemness of NSPCs in a slowly proliferating stem cell state by repressing the cell cycle regulator cyclin D1 during cortex development [19]. When NSPCs enter the stage of differentiation, the levels of SOX2 decrease, which releases this repression and thus promotes cell cycle re-entry and NPC proliferation [20]. In this study, the SOX2 staining results showed that the number of SOX2 cells in SVZ gradually decreased during neurodevelopment. Notably, most cells with high level of H3K27me3 showed high level of SOX2, whereas H3K36me3 cells presented low co-staining with SOX2 cells. Furthermore, H3K4me3 and SOX2 co-staining are rare in SVZ. There is a positive correlation between the expression of SOX2 and stemness of NSPCs [21]. Thus, our results define histone methylations specific for SOX2-positive NSPCs. Moreover, we reveal that high levels of H3K27me3 exist in the early stage of NSPC development; H3K36me3 is characteristic of metaphase while H3K4me3 is enriched in the mid and later stages of NSPC development.

In the mammalian embryo brain, the proliferative region comprises two distinct zones: VZ, which is a neuroepithelial layer directly adjacent to the ventricular lumen, and SVZ, which is positioned superficial to the ventricular zone [22] (Fig. 1a). Radial glial cells (RGCs, one type of embryonic neural stem cells) reside in the VZ and generate both intermediate progenitor cells (IPCs, one type of embryonic neural precursor cells) and cortical neurons. IPCs migrate away from the ventricular surface and establish the SVZ [23]. Therefore, the cellular composition is different in VZ and SVZ. RGCs are mostly concentrated in VZ and most of IPCs located in SVZ. In this study, embryo brain staining showed high levels of H3K27me3 and H3K36me3 in VZ, and of H3K4me3 in SVZ. Thus, it suggests high level of H3K27me3 and H3K36me3 at early stage of embryonic neural stem cell development, and of H3K4me3 at middle/late stage.

Further, our results identify significant differences among immunocytochemical double labeling in the P10 SVZ. However, we found that these distinct features disappeared in 2 months or E18; the number of NSPCs in SVZ was significantly decreased during neuronal development, and the dynamics of histone methylations described here might be one of the mechanisms underlying this regulation and might encode the difference between embryonic NSPCs and adult NSPCs. One major difference between adult and embryonic neural stem cells is their different number and their ability to differentiate into various cell types. Embryonic NSPCs can divide asymmetrically to generate neurons directly or indirectly through intermediate progenitor cells and oligodendrocytes. More importantly, at the end of the embryonic development, embryonic NSPCs begin to detach from the apical side and convert into astrocytes. Even if adult NSPCs can continue to generate neurons and oligodendrocytes, they cannot differentiate into astrocytes [24]. Histone methylation introduces epigenetic modifications with close ties to transcription and has been directly linked to lifespan regulation in many organisms [25]. For example, upon differentiation towards the neuronal lineage, some bivalent genes became expressed and lost the H3K27me3 mark, whereas those that were silenced lost the H3K4me3 and retained H3K27me3 [26]. Therefore, it is not unlikely that the embryonic and adult NSPC states are maintained by differential histone methylation profiles.

Chromatin, the template for epigenetic regulation, is a highly dynamic entity that is constantly reshaped during neurodevelopment [27]. Epigenetic regulation by histone methylation provides the necessary plasticity for cells to respond to environmental and positional cues, enabling the maintenance of acquired information without changing the DNA sequence. In this study, we showed different subtypes of NSPCs represented different features of histone methylations. These results may reveal novel insight into the onset of neurodevelopment and provide an innovative epigenetic signature for discovery and characterization of key regulatory genes for neurogenesis. However, further studies, especially whole epigenome analysis and histone profiling, are necessary for in-depth understanding of the role for individual histone methylation domains in neurodevelopment.

## Electronic Supplementary Material

ESM 1 (DOCX 14 kb)

ESM 2 (JPG 4704 kb)
