# Supplementary material for: Histone Methylations Define Neural Stem/Progenitor Cell Subtypes in the Mouse Subventricular Zone
Source: Mol Neurobiol. 2019 Oct 25;57(2):997–1008. doi: 10.1007/s12035-019-01777-5 (PMC7031420; doi:10.1007/s12035-019-01777-5)
Supplement: Supplementary file 1 — (DOCX 14 kb) [file 12035_2019_1777_MOESM1_ESM.docx]

**Supplement figure legends**

**Supplement Fig. 1**

H3K27me3, H3K36me3 and H3K4me3 co-stained with SOX2 during neurodevelopment in SGZ. Immunofluorescent staining showed that high level of H3K36me3 and H3K4me3 co-stained with SOX2 at E18 (A), P10 (B) and 2M (C), while H3K27me3 SOX2 double positive cells were just sporadic at any time-point. Nuclei were counterstained with DAPI. E18: embryo at day 18; P10: postnatal at day 10; 2M: adults 2 months. Scale bar = 50 μm. (D) The number of immunolabeled cells was counted for three sections in each mouse and each value represents the mean ± SD of three mice (n=3). **P* < 0.05, ***P* < 0.01, ****P* < 0.001 versus H3K27me3 group; ^#^P < 0.05 versus H3K36me3 group.
