# Supplementary figures and images for: Histone Methylations Define Neural Stem/Progenitor Cell Subtypes in the Mouse Subventricular Zone
Source: Mol Neurobiol. 2019 Oct 25;57(2):997–1008. doi: 10.1007/s12035-019-01777-5 (PMC7031420; doi:10.1007/s12035-019-01777-5)

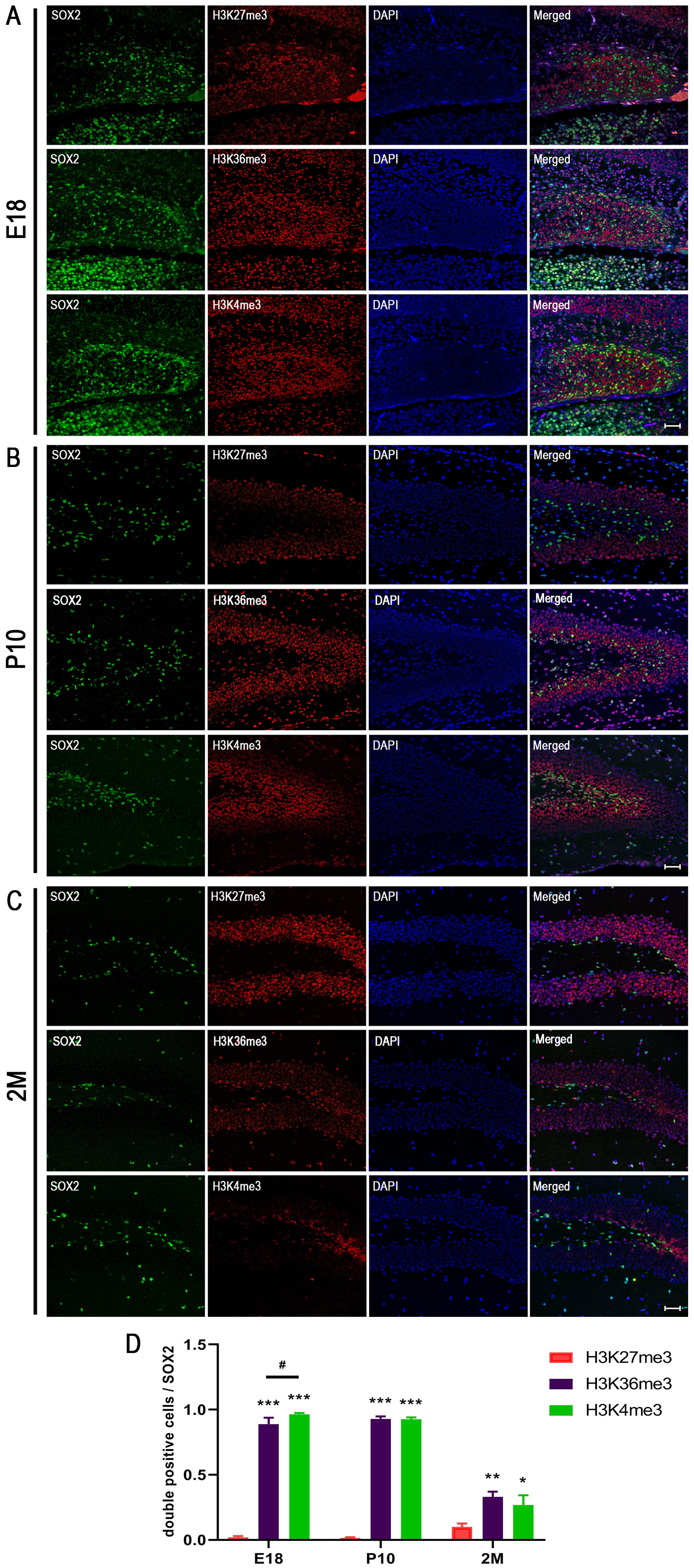

Supplement: Supplementary file 2 — (JPG 4704 kb) [file 12035_2019_1777_MOESM2_ESM.jpg]
